# Preclinical Retinal Disease Models: Applications in Drug Development and Translational Research

**DOI:** 10.3390/ph18030293

**Published:** 2025-02-21

**Authors:** Sudha Priya Soundara Pandi, Hanagh Winter, Madeleine R. Smith, Kevin Harkin, James Bojdo

**Affiliations:** 1 Medinect Bioservices Ltd., Belfast BT7 1NF, UK; sudha@medinect.co.uk (S.P.S.P.); hanagh@medinect.co.uk (H.W.); maddie@medinect.co.uk (M.R.S.); kevin@medinect.co.uk (K.H.); 2 Wellcome-Wolfson Institute for Experimental Medicine, Queen’s University Belfast, Belfast BT9 7BL, UK

**Keywords:** translational drug development, retinal disease models, systemic disease application, preclinical research

## Abstract

Retinal models play a pivotal role in translational drug development, bridging preclinical research and therapeutic applications for both ocular and systemic diseases. This review highlights the retina as an ideal organ for studying advanced therapies, thanks to its immune privilege, vascular and neuronal networks, accessibility, and advanced imaging capabilities. Preclinical retinal disease models offer unparalleled insights into inflammation, angiogenesis, fibrosis, and hypoxia, utilizing clinically translatable bioimaging tools like fundoscopy, optical coherence tomography, confocal scanning laser ophthalmoscopy, fluorescein angiography, optokinetic tracking, and electroretinography. These models have driven innovations in anti-inflammatory, anti-angiogenic, and neuroprotective strategies, with broader implications for systemic diseases such as rheumatoid arthritis, Alzheimer’s, and fibrosis-related conditions. By emphasizing the integration of the 3Rs principles and novel imaging modalities, this review highlights how retinal research not only enhances therapeutic precision but also minimizes ethical concerns, paving the way for more predictive and human-relevant approaches in drug development.

## 1. Introduction

Drug development is a rigorous, multi-phase process designed to ensure the safety, efficacy, and quality of a therapeutic candidate. It begins with the discovery phase, where potential drug candidates are identified through screening and preclinical studies, including mechanism of action (MoA) studies to elucidate how the drug interacts with its biological target. From a regulatory standpoint, MoA studies provide critical data supporting the scientific rationale for a drug’s use in specific conditions. These studies are followed by efficacy and safety evaluations in preclinical models, including non-human primate studies, conducted in adherence with regulatory guidelines such as Good Laboratory Practice (GLP) where applicable. Comprehensive documentation of these studies is required to meet the standards set by regulatory bodies like the Food and Drug Administration (FDA) or European Medicines Agency (EMA), forming the basis for Investigational New Drug (IND) applications and enabling the transition to clinical trial.

The principles of the 3Rs—Replacement, Reduction, and Refinement—first described by Russell and Burch in 1959 [1], are designed to minimize the use of animals in research by promoting alternative methods, reducing the number of animals required, and improving experimental protocols. Notably, some regulatory approvals, such as specific vaccine developments during the COVID-19 pandemic, have relied heavily on in vitro and computational models, with a view to bypass certain animal studies entirely [2,3]. Advances in 3D cell culture systems, including organoids and microfluidic organ-on-chip platforms, offer significant promise for replicating human physiology with greater accuracy. However, animal studies remain essential for now, as these 3D systems often lack the ability to fully recapitulate the complexity of whole-organ interactions, systemic responses, and longitudinal disease progression. Until 3D technologies are fully validated and widely integrated into regulatory frameworks, animal models will continue to play a critical role in capturing the broader physiological context necessary for drug development.

While animal studies remain essential for understanding complex biological systems, they have limitations, particularly in non-GLP studies, where variability, lack of full human disease pathology and lack of standardization can reduce reliability. Statistics highlight a significant translational gap: only about 10–12% of drugs that show efficacy in preclinical models successfully reach approval after human clinical trials [4], underscoring the need for more predictive and human-relevant testing approaches.

The retina is a prototypical immune-privileged tissue, safeguarded from external and internal insults by its highly efficient blood–retina barriers (BRBs) and a unique immune-suppressive microenvironment [5]. This immune privilege is maintained through strategies of avoidance, tolerance, and resistance. The retina avoids immune activation by limiting exposure to systemic inflammatory and immunogenic disturbances, while its tolerance mechanisms actively suppress inflammation to protect its delicate structure and function [6]. Additionally, the retina’s own defense systems, including macroglia (Müller cells and astrocytes), microglia, and the complement system, work to resolve threats and maintain homeostasis, particularly under mild or transient conditions. However, when these mechanisms are breached—due to injury [7,8], aging [6], or diseases [9]—the immune privilege can be disrupted, triggering para-inflammatory responses observed in aging, which eventually progress to chronic inflammation in disease conditions.

This dual nature of the retina makes it an excellent organ for testing advanced therapies, such as gene and cell-based treatments. Its immune privilege reduces the risk of systemic immune responses, while its localized immune activity provides a controlled environment to monitor therapeutic efficacy and potential adverse effects. In some cases, retinal disease models intentionally disrupt immune privilege, as seen in models of uveitis or age-related macular degeneration (AMD). These models provide a unique opportunity to evaluate therapeutics aimed at modulating immune responses or treating inflammatory conditions.

In addition to immune-privileged systems, the retina features a complex arrangement of neurons and blood vessels. The retinal endothelial cells and the choroid supply essential nutrients and oxygen to the retina. The retinal endothelial cells, along with the retinal pigment epithelium (RPE), play a vital role in maintaining the BRB, ensuring the retina’s microenvironment remains stable. Much like the immune system, the vascular system of the retina relies on a delicate balance of pro-angiogenic, and anti-angiogenic factors secreted by retinal cells to sustain normal blood vessel function. Factors such as aging, environmental influences, and genetic predispositions can disturb this balance, leading to abnormal blood vessel growth, which is seen in conditions like AMD and diabetic retinopathy [10,11]. Choroidal neovascularization and diabetic retinopathy preclinical models provide human AMD and DR pathological features to evaluate therapeutics such as anti-angiogenic agents aimed at treating microaneurysms and angiogenesis.

As well as its vascular and immune intricacies, the retina is distinguished by its advanced neuronal network, often referred to as the “window to the brain”. Photoreceptor cells in the retina gather visual information and relay signals through a well-organized neuronal network to the brain. However, this network is susceptible to various challenges, including aging, oxidative stress, low oxygen levels, inflammation, and genetic influences. These challenges can lead to neuronal death, impairing image processing and resulting in distorted visual perception [12,13]. This type of pathology is evident in diseases such as atrophic AMD, retinitis pigmentosa (RP), and glaucoma, all of which are marked by progressive vision loss due to neuronal degeneration. Various systemic neurodegenerative preclinical models such as MPTP, Alzheimer’s, and glaucoma models provide an excellent model to test the neuroprotective agent on its own or in combination with an anti-inflammatory or anti-angiogenic agent to treat the neurodegeneration.

**Figure 1:**
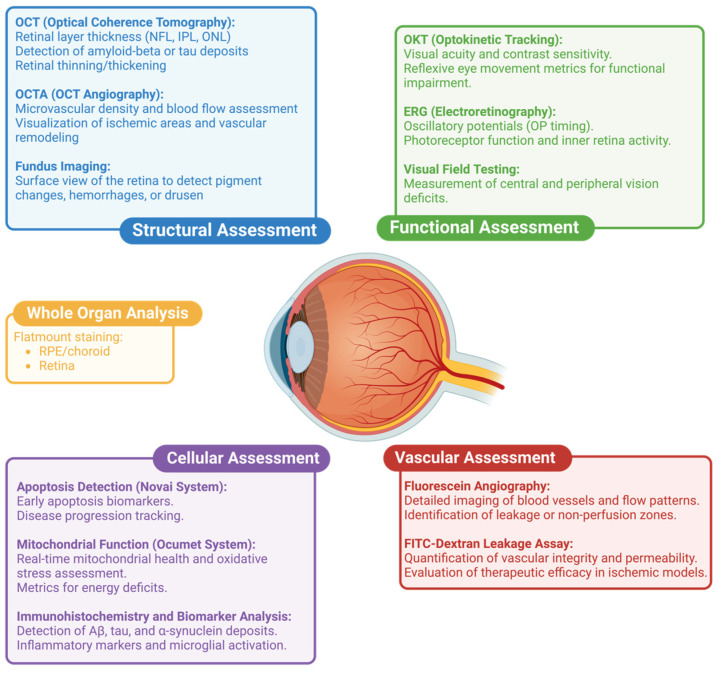
The eye—a window into disease. The eye’s unique accessibility and transparency make it ideal for advanced imaging and, as such, retinal assessment leads the way in imaging sophistication. Some of these tools are showcased above, including structural (blue), functional (green), cellular and molecular assessments (purple), vascular (red), and whole organ analyses (yellow). By combining these modalities, retinal imaging offers unparalleled insights into structure, function, and disease progression. Figure created in http://www.BioRender.com (accessed on 2 December 2024).

The eye’s accessibility for in vivo imaging enables real-time visualization of therapeutic effects on both structural and functional readouts, leveraging a suite of advanced tools. Optical coherence tomography (OCT) provides detailed structural analysis of retinal layers [14], while OCT angiography (OCT-A) visualizes microvascular changes and blood flow [15]. Electroretinography (ERG) captures functional assessments of retinal activity, revealing deficits in photoreceptor and ganglion cell function [16]. Fundus imaging allows for the evaluation of surface-level changes, such as drusen [17], pigmentary alterations [18], and retinal hemorrhages [19]. Fluorescein angiography and fundus fluorescein angiography (FFA) assess vascular leakage and integrity, crucial for studying diseases involving BRB breakdown [20]. Confocal scanning laser ophthalmoscopy (cSLO) adds another dimension by enabling high-resolution imaging of fluorescently labeled markers, providing insights into protein aggregation, specific marker expression, and cell enumeration/activation [21], for example, for studying disease-susceptible but harder-to-image retinal ganglion cells [22]. Measurement of the optokinetic reflex offers functional measures of visual acuity and contrast sensitivity, contributing to a comprehensive understanding of therapeutic effects [23] (Figure 1).

In addition, the eye presents a unique opportunity for drug developers, offering both localized and systemic delivery options to effectively target diseases. Localized intraocular delivery methods, such as intravitreal (IVT) and subretinal or suprachoroidal injections, allow for direct therapeutic application to the organ and site of damage, ensuring high concentrations at the disease locus. Systemic administration can also be leveraged due to the eye’s highly vascularized tissue, which facilitates exposure, especially in conditions where the BRB is compromised, as is the case in most retinal disease models. Emerging roles for topical therapies and advanced ocular drug delivery techniques, such as nanoparticle-based systems, sustained-release implants, and microneedles, are expanding the therapeutic arsenal. These innovations aim to enhance drug penetration, improve patient compliance, and reduce the burden of repeated invasive procedures, paving the way for a new era of precision and convenience in ocular treatment.

While in vivo models are not without limitations, when designed and executed rigorously they can replicate complex biological interactions that are difficult to achieve in vitro, providing invaluable insights for drug development. The eye offers significant advantages in this context, enabling comprehensive whole-organ analysis and the study of diverse pathological etiologies, including angiogenesis, fibrosis, inflammation, hypoxia, and oxidative stress.

The aim of this review is to highlight the translational relevance of retinal disease models in drug development, both for ocular conditions and broader systemic diseases. By emphasizing the retina’s unique combination of functional, structural, and molecular readouts, we demonstrate how these models can bridge the translational gap while adhering to the ethical principles of the 3Rs. This review explores the potential of retinal models to advance treatments, improve predictive accuracy, and reduce the need for multiple disease-specific studies, thereby maximizing the impact of each preclinical investigation.

## 2. Animal Models in Retinal Disease Research and Applications

### 2.1. Anti-Inflammatory Drug Targets

Inflammation is a vital process for maintaining homeostasis, but its dysregulation can drive pathological conditions. The two primary forms of inflammation—acute and chronic—have distinct roles in health and disease. Acute inflammation is an immediate, protective response to injury or infection, aimed at restoring tissue integrity. Conversely, chronic inflammation results from prolonged immune activation, contributing to the progression of conditions such as cancer, cardiovascular diseases like myocarditis and pericarditis, liver diseases such as autoimmune hepatitis, chronic obstructive pulmonary disease (COPD), chronic kidney disease, type II diabetes, Alzheimer’s and Parkinson’s diseases, systemic lupus erythematosus, and rheumatoid arthritis [24].

Among the key drivers of these inflammatory processes are microglia and macrophages, which play pivotal roles in initiating and modulating inflammation [25,26,27]. Microglia, the resident immune cells of the central nervous system, including the retina, dynamically transition between pro-inflammatory and anti-inflammatory states [28]. This duality allows them to mediate tissue repair or exacerbate damage, depending on the context [29]. Similarly, macrophages infiltrate tissues during inflammation, contributing to both the onset and resolution of immune responses [30,31]. In the retina, chronic activation of microglia and recruitment of macrophages are significant contributors to the pathology of diseases like diabetic macular edema [32,33], retinal degeneration [34], uveitis [35], and retinitis pigmentosa [18,36]. In addition to microglia and macrophages, macroglia such as Müller glia and astrocytes also play crucial roles in retinal inflammation. Müller cells, which extend across the entire thickness of the retina, become reactive when under stress, releasing inflammatory cytokines and contributing to gliosis/scar formation, breakdown of the BRB, and neurodegeneration [37,38]. Astrocytes, mainly found in the retinal nerve fiber layer, support retinal ganglion cells and help maintain the BRB. However, during pathological conditions, reactive astrocytes release inflammatory mediators that can worsen retinal damage, including degeneration of the optic nerve [39,40]. Activation of Müller cells and astrocytes significantly contributes to the pathogenesis of AMD [41], DME [42,43], DR [44,45], RP [40], and glaucoma [46].

The most used agents to induce and study inflammation in the animal model for systemic drug development are lipopolysaccharide (LPS) and ovalbumin (OVA). For example, APoE deficient mice and TNF-α knockout mice injected with LPS are used to investigate the inflammatory plaques in atherosclerosis and neuroinflammation observed in the brain, respectively [47,48]. Similarly, ovalbumin systemically injected in the BALB/c mice induces chronic lung inflammation, mimicking asthma [49,50,51]. However, these models have limitations, primarily due to the end-stage detection of inflammation in the corresponding tissues ex vivo, which prevent the comprehensive understanding of early detection and in vivo longitudinal monitoring during disease progression or therapeutic intervention.

**Figure 2:**
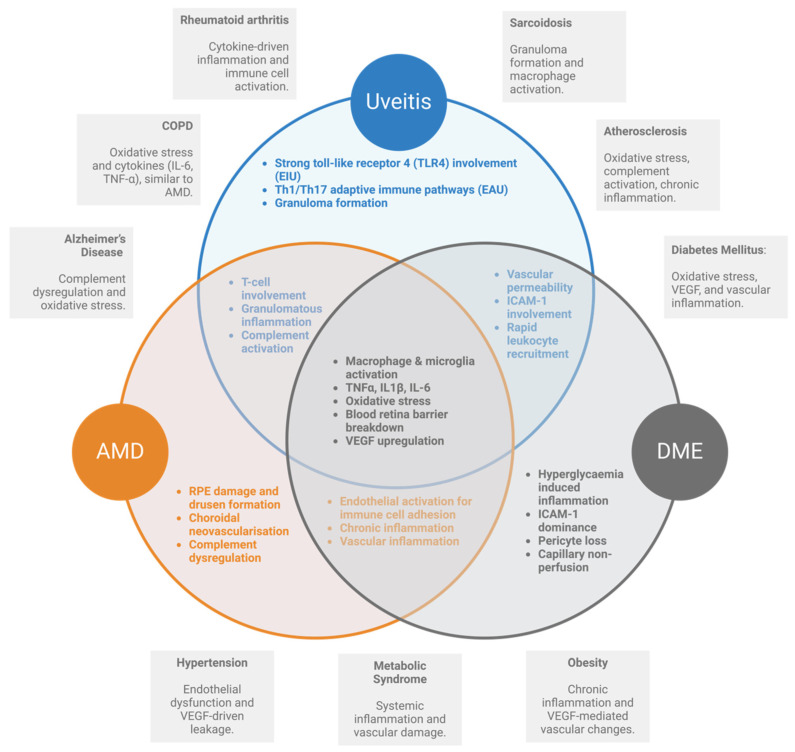
Translational Insights from Retinal Inflammation Models. There is significant overlap in inflammatory mechanisms between retinal diseases including age-related macular degeneration (AMD), diabetic macular edema (DME), and uveitis, as well as with systemic inflammatory diseases. Central shared mechanisms include cytokine-driven inflammation, oxidative stress, immune cell activation, complement activation, and vascular inflammation. Retinal models such as endotoxin-induced uveitis (EIU), experimental autoimmune uveitis (EAU), AMD models, and DME models illustrate how preclinical studies capture these pathways, bridging insights to systemic conditions like rheumatoid arthritis, inflammatory bowel disease, multiple sclerosis, atherosclerosis, and systemic lupus erythematosus. This overlap emphasizes the translational relevance of retinal research in advancing systemic anti-inflammatory therapies. Figure created in http://www.BioRender.com (accessed on 2 December 2024).

The eye offers unique advantages as a site for preclinical drug testing, particularly for anti-inflammatory therapies targeting microglia and macrophages. Retinal disease models enable real-time, in vivo evaluation of disease mechanisms and therapeutic effects, providing insights into multifaceted processes (Figure 2). As shown in Figure 2, Uveitis, AMD, and DR share overlapping pathological inflammatory responses including BRB breakdown, inflammatory cytokines release, vascular leakage, and neuronal damage. Especially, chronic inflammation in uveitis contributes to CNV in AMD [52] and fluid accumulation in DME [53]. Unlike systemic models, retinal models, especially inflammation models outlined in Figure 2, facilitate dynamic assessment of drug effects, and the eye’s accessibility allows for whole-organ analysis, offering a detailed understanding of tissue-level responses. For instance, in the experimental autoimmune uveitis (EAU) model, EAU scoring is typically performed using in vivo fundoscopy, which provides an overall readout of inflammation. However, OCT offers the advantage over fundoscopy by enabling the detection of early inflammatory cell infiltrate at the retinal/choroidal junction. OCTA used in these models provides insights into the vasculitis [54,55,56]. Similarly in AMD animal models such as the sodium iodate model and choroidal neovascularization (CNV) model, the inflammatory cell infiltrates are observed as hyperreflective foci in the OCT images in vivo [57,58,59]. In diabetic edema (DME) models, the inflammatory pathology of leukocyte accumulation was monitored in vivo by acridine orange leukocyte fluorography [60]. From a 3R standpoint, inflammation models offer the longitudinal study framework to help reduce the number of animals used for this study and enhance the depth of data collected.

This advantage has been pivotal in the development of drugs targeting inflammatory pathways, with several examples demonstrating the translational potential of ocular drug testing. For instance, Aviceda Therapeutics’ AVD-104, a glycan-coated nanoparticle engineered to target Siglecs, self-recognition receptors expressed on retinal immune cells such as macrophages and microglia, modulates inflammation through ITIM signaling, offering a novel therapeutic approach for dampening retinal immune responses [61].

Other examples include drugs targeting complement pathways, initially trialed for AMD, that have progressed to treating diseases such as paroxysmal nocturnal hemoglobinuria (PNH) and atypical hemolytic uremic syndrome (aHUS). Notable examples include Eculizumab (Soliris) developed by Alexion Pharmaceuticals, a monoclonal antibody that inhibits complement component C5. While it was explored for AMD treatment, it gained approval for PNH and aHUS, effectively reducing hemolysis and thrombotic microangiopathy in these conditions [62]. Ravulizumab (Ultomiris), also developed by Alexion Pharmaceuticals, is a long-acting C5 inhibitor investigated for AMD and subsequently approved for PNH and aHUS, offering extended dosing intervals compared to eculizumab [62]. Pegcetacoplan (Empaveli/Syfovre), developed by Apellis Pharmaceuticals, is a C3 inhibitor initially studied for AMD before it received approval for PNH and, more recently, for geographic atrophy secondary to AMD [63]. The success of complement inhibitors in rare diseases like PNH and aHUS provides a strong foundation for their use in chronic diseases. However, understanding the specific complement pathways involved and the long-term effects of systemic complement inhibition will be essential for their successful application in broader conditions.

FDA-approved drugs for systemic conditions have also been explored in the EAU model. For example, Teriflunomide, approved for multiple sclerosis, reduced clinical and histopathological EAU scores by suppressing dendritic cells, Th1/Th17 differentiation, and cytokine production (TNF-α, IFN-γ, IL-17) [64]. Apremilast, approved for psoriatic arthritis, demonstrated anti-inflammatory effects by inhibiting Th17 cells and increasing Tregs via PI3K/AKT/FoxO1 signaling pathway, helping maintain retinal homeostasis [65].

While systemic anti-inflammatory drugs have been tested in retinal models, there remains significant potential for further exploration. Anti-TNF agents such as adalimumab and infliximab, which are FDA-approved for rheumatoid arthritis and Crohn’s disease [66,67], can be evaluated in retinal models for conditions like uveitis and DME. Similarly, IL-6 inhibitors like tocilizumab, used in systemic inflammatory diseases, may also target common pathways in retinal inflammation, particularly in conditions involving cytokine storms [68]. Additionally, JAK inhibitors, which have shown promise in systemic immune disorders, could also present an opportunity for targeting retinal inflammation, further expanding the therapeutic potential of these agents [69]. The summary of the above-mentioned drugs and their efficacy is outlined in Table 1.

### 2.2. Wound Healing Mechanisms and Their Pathological Transformation in Retinal Diseases

**Figure 3:**
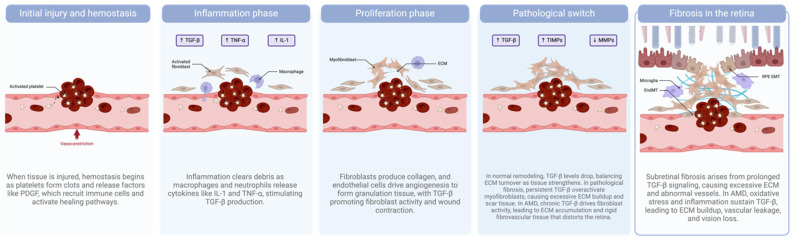
From injury and hemostasis through inflammation and proliferation, retinal repair mechanisms aim to restore tissue integrity. However, sustained inflammation and TGF-β activation (releasing cytokines like IL-1 and TNF-α) can drive a pathological switch, leading to a tissue inhibitor of metalloproteinase (TIMP)/MMP imbalance, excessive ECM deposition, neovascularization, and ultimately fibrosis. In AMD, subretinal fibrosis is characterized by fibrovascular tissue formation, chronic inflammation, ECM remodeling, and vascular leakage. Figure created in http://www.BioRender.com (accessed on 2 December 2024).

Wound healing is a highly orchestrated biological process involving multiple stages: hemostasis, inflammation, proliferation, and remodeling [70]. During these stages, various cellular and molecular mechanisms are activated, including the regulation of angiogenesis, extracellular matrix (ECM) remodeling, and immune cell recruitment. Under normal conditions, these processes restore tissue integrity. However, dysregulation of these mechanisms can result in sustained fibrovascular membranes and ultimately scarring along with chronic inflammation (Figure 3) [71]. In retinal diseases, such as neovascular AMD, the wound healing response can turn pathological, contributing to vision loss [72].

Anti-angiogenic therapies were first developed for cancer treatment, targeting the overexpression of vascular endothelial growth factor (VEGF) to inhibit tumor angiogenesis [73]. Bevacizumab (Avastin), an anti-VEGF monoclonal antibody, was initially approved for metastatic colorectal cancer [74] and later applied off-label for retinal diseases like AMD and DME [75]. These drugs effectively suppress neovascularization, a hallmark of both tumors and retinal diseases. Subsequently, Ranibizumab (Lucentis) [76] and Aflibercept (Eylea) [77] were developed specifically for retinal conditions, becoming mainstays for AMD and DME management. Despite their efficacy in reducing neovascularization, these agents do not address the fibrotic component of diseases like AMD, leaving subretinal fibrosis an unmet therapeutic need.

Preclinical models such as the CNV model and the oxygen-induced retinopathy (OIR) model have been instrumental in evaluating anti-angiogenic therapies and elucidating mechanisms of angiogenesis. The CNV model simulates neovascularization driven by VEGF, mimicking pathological conditions in AMD. This model has been widely used to assess the efficacy of anti-VEGF agents, including Ranibizumab and Aflibercept [4,78]. Similarly, the OIR model replicates ischemia-induced angiogenesis seen in conditions like retinopathy of prematurity (ROP) and diabetic retinopathy. The OIR model provides robust endpoints for studying pathological neovascularization, making it an essential tool for investigating new therapeutic targets and optimizing anti-angiogenic strategies [79]. Together, these models offer valuable insights into angiogenic processes and therapeutic interventions for both retinal and systemic vascular diseases.

Fibrosis occurs when wound healing mechanisms become dysregulated, leading to excessive ECM accumulation and the activation of fibrogenic pathways involving transforming growth factor-beta (TGF-β), platelet-derived growth factor (PDGF), and other signaling molecules [80]. In retinal diseases, subretinal fibrosis contributes significantly to vision loss in conditions like neovascular AMD [72], where fibrosis is increasingly recognized as a wound healing-related disease. Subretinal fibrosis, a late-stage complication of neovascular AMD, results from chronic inflammation and recurrent damage to the retinal pigment epithelium and photoreceptors [81]. This process involves fibrovascular tissue formation [82], ECM remodeling [83], and vascular leakage [84], exacerbating vision impairment.

The development of sustained fibrosis models, such as the two-stage laser-induced choroidal neovascularization (liCNV) model, provides a platform to study chronic fibrotic processes and evaluate therapeutic interventions. Unlike traditional systemic fibrosis models, the subretinal fibrosis model offers distinct advantages from both scientific and ethical perspectives. It effectively replicates the chronicity of human fibrosis, maintaining fibrotic responses for over 42 days [85]. Mechanistically, it shares key fibrotic pathways with systemic conditions, enabling the study of therapeutic agents relevant to a range of fibrotic diseases. From an animal welfare and 3R perspective—refinement, reduction, and replacement—the subretinal fibrosis model is significantly advantageous. It avoids the invasive surgical procedures or systemic toxic exposures often required in other fibrosis models, reducing animal suffering. Additionally, retinal imaging techniques like OCT and fundus photography allow for non-invasive, longitudinal assessments, minimizing the need for large animal cohorts and aligning with the principles of reduction.

Whilst CNV induction by laser photocoagulation of the choroid is the most widely used technique in CNV models, numerous other methods of induction have been reported. These include surgically induced CNV lesions through disruption of the Bruch’s membrane mechanically or immunologically, where models have employed subretinal injections of various pro-angiogenic agents, such as recombinant adenovirus vectors overexpressing VEGF [86,87], or subretinal Matrigel deposition [88]. Additionally, completely non-invasive models of CNV have been described that utilize transgenic and knockout mouse lines that spontaneously develop CNV lesions. For example, the JR5558 transgenic mouse harbors mutations in *Crb1^rd8^ and Jak3^m1J^*, which cause a neovascular phenotype of CNV and retinal degeneration [89,90,91]. Many of these methods, however, have produced considerably lower success rates of CNV lesion induction compared to laser-induced CNV models, suggesting that laser induction provides a more reliable model for higher throughput drug screening studies [92].

Several drugs have shown promise in targeting fibrosis in retinal diseases, with some crossing over to systemic fibrotic and wound healing indications. Dexamethasone, a corticosteroid, exerts anti-inflammatory and anti-fibrotic effects through cytokine and VEGF inhibition [93]. Clinically, it is approved for DME [94] and retinal vein occlusion [95], while also being widely used in systemic inflammatory and fibrotic diseases like rheumatoid arthritis and asthma. Pirfenidone, an antifibrotic agent that inhibits TGF-β signaling and ECM production, has demonstrated efficacy in preclinical retinal disease models of post-traumatic proliferative vitreoretinopathy and CNV [96,97]. It is currently approved for idiopathic pulmonary fibrosis (IPF) [98] and is under investigation for other systemic fibrotic diseases. AVD-104, initially developed for geographic atrophy in AMD, has demonstrated antifibrotic effects and is now being explored for systemic applications such as myelofibrosis, illustrating its translational potential.

Given the interplay between angiogenesis and fibrosis in retinal diseases, combining anti-VEGF agents with emerging antifibrotic therapies offers a promising strategy to improve outcomes. Sustained fibrosis models, such as the subretinal fibrosis model, have enabled the preclinical evaluation of such approaches. For instance, the combination of anti-VEGF agents with compounds like Pirfenidone or integrin inhibitors holds the potential to address both neovascularization and fibrotic scarring. These approaches not only improve efficacy in retinal diseases but also set the stage for translating retinal therapies to systemic fibrotic and wound healing conditions, advancing the treatment landscape for chronic fibrotic diseases. By integrating sustained models that prioritize animal welfare and align with the 3Rs, researchers can accelerate therapeutic discovery while minimizing ethical concerns.

### 2.3. Preclinical Models of Hypoxia-Associated Disease

Hypoxia is a condition where insufficient oxygen reaches the surrounding tissues. The hypoxia-inducible factor (HIF) is crucial for managing how cells respond to low oxygen levels [99]. HIFs are transcription factors that stabilize under low-oxygen conditions, initiating the transcription of genes involved in oxygen adaptation, including those regulating angiogenesis (via VEGF), metabolism, and cell survival [99]. Given the importance of the tightly organized vasculature in the eye, HIFs play a crucial role in maintaining its normal function or development; however, its expression is not ubiquitous across the different cell types present in the eye [100]. As such, it has been shown through conditional deletions of HIF-1α in the developing mouse retina that its expression in RPE and Müller cells, but not in astrocytes or photoreceptors, significantly contributing to normal development and function [101,102,103,104].

While these mechanisms are adaptive, prolonged hypoxia often leads to pathological outcomes, such as aberrant vascular growth and fibrosis. Subsequently, hypoxic conditions are often linked to retinal diseases like diabetic retinopathy, AMD, and glaucoma [105]. In diabetic retinopathy, hypoxia occurs due to damage to blood vessels, which leads to poor oxygen delivery and increased metabolic needs in the retina, which is accompanied by activation of HIF-1α in both photoreceptor and Müller glial cell types, as well as in astrocytes [105,106]. In AMD, hypoxia is caused by decreased blood flow in the choroid, thickening of Bruch’s membrane, higher oxygen demands, and ineffective oxygen supply from new blood vessels, where HIF-1α activation in RPE or photoreceptor cells has been shown for both dry and wet AMD [107,108]. In glaucoma, hypoxia mainly results from reduced blood flow and increased intraocular pressure (IOP), which together hinder oxygen delivery to retinal ganglion cells (RGCs) and the optic nerve head, increasing levels of HIF-1α [109].

Hypoxia remains a central therapeutic target in both retinal and systemic diseases due to its role in driving angiogenesis, inflammation, and fibrosis. Experimental models like OIR and ischemia-reperfusion (I/R) injury, i.e., Carotid Artery Occlusion (CAO) and Retinal Vein Occlusion (RVO) models, provide robust platforms to evaluate new interventions, particularly those addressing the complex molecular interplay of hypoxia and its downstream effects. These models not only advance our understanding of retinal hypoxia but also offer valuable insights into systemic diseases such as COPD, stroke, peripheral artery disease, and myocardial infarction (MI). By combining HIF-targeted therapies with existing agents like anti-VEGFs, researchers can explore promising avenues for improving outcomes in ischemia- and hypoxia-driven diseases across multiple organ systems.

Numerous preclinical models are used to study hypoxia-associated diseases, each with distinct strengths and limitations, summarized in Table 2. For example, the hindlimb ischemia model is commonly employed for studying peripheral artery disease and involves ligation or excision of blood vessels to induce ischemia [110]. While this model provides insights into vascular remodeling and angiogenesis, variability in surgical techniques often compromises reproducibility, and the invasive nature of the procedures raises ethical concerns. Similarly, MI models, achieved through coronary artery ligation, are indispensable for studying ischemic heart disease but require highly invasive interventions and extensive post-surgical care, posing significant challenges to animal welfare [111]. Systemic models such as hypoxia chambers, which create generalized low-oxygen conditions, lack the ability to replicate the localized ischemia and subsequent revascularization processes that are central to many human diseases.

Through multiple methods, researchers can induce hypoxia and ischemic injury in the retina. The retinal arterial occlusion (RAO) and the RVO model create a vascular blockade, leading to retinal ischemia. Both models, when carried out by experienced researchers, produce highly reproducible data with the added advantage of allowing real-time retinal imaging to monitor key morphological changes in vivo. Utilizing OCT-A as a tool to track blood flow, and even glucose and oxygen levels in individual vessels, is particularly powerful in this scenario [112,113,114]. The most translatable disease this can be related to is Acute Ischemic Stroke where MRI studies have shown that 13–24% of retinal arterial occlusion patients have silent cerebral infarcts, emphasizing the strong association between retinal ischemia and stroke risk [115,116,117]. Furthermore, the bilateral common carotid artery occlusion (BCAO) rat model, which involves ligating the carotid artery and branches before the ophthalmic artery, causes an infarcted region in the brain and retina [118,119]. Studies also show the synergistic effects in humans [120,121,122]. Many similarities between the retina and brain ischemia occur, such as the initial penumbra–core model that supersedes the ischemia, the intricate interplay in the neovascular unit, and the similarities in the BRB dysfunction and the blood–brain barrier breakdown observed in AIS, which leads to vascular leakage neuroinflammation and secondary neuronal loss. The strong evidence showing the similarities between retinal ischemia in the BCAO model when compared to the brain suggests that other models such as the retinal arterial occlusion model and the RVO model are appropriate models for investigating cerebrovascular research. Specifically, BCAO and RAO models closely mimic large vessel diseases, while RVO serves as a model for small vessel diseases, reflecting the pathophysiology of different types of stroke and microvascular ischemic conditions [118].

**Table 2:** Preclinical models to study hypoxia-related diseases.

Model	Disease	Induction Method	Readouts	Limitations	Therapies Tested in Model
Hindlimb ischemia	Peripheral artery disease	Femoral artery ligation/excision	Blood flow (Laser Doppler), limb function, clinical scoring, histology, biomarkers	Variability in surgical technique, acute nature, limited functional assessment, species difference, ethical considerations	IL-33 [123], Sotagliflozin [124], Shuxuetong injection [125]
Myocardial infarction	Ischemic heart disease	Coronary artery ligation	ECG, echocardiography, blood pressure monitoring, infarct size, histology, biomarkers	Surgically complex, high perioperative mortality, limited chronic remodeling in rodents, ethical considerations	Mir-155 [126], Baicalin [127], Curcumin [128]
Ischemia-reperfusion injury	DR, glaucoma, RVO	Temporary occlusion of artery prior to reperfusion	ERG, IOP measurements, visual acuity and behavioral tests, OCT, FA, histology, biomarkers	Acute injury, reperfusion variability, species differences	Edaravone [129], Metformin [130], Peptain-1 [131], Resveratrol [132,133,134], DMHCA [135]
Oxygen-induced retinopathy	DR, AMD, ROP	Hyperoxia (75% O_2_) followed by normoxia	Retinal neovascularization, vessel density, ERG, visual acuity and behavioral tests, OCT-A, FA, histology, biomarkers	Species-dependent differences, neonatal model limitations, spontaneous regression in mice	Anti-VEGF therapeutics [136,137,138], Heparin derivatives [139], NP-G2-044 [140], Sorafenib [141], Metformin [142], Resveratrol [143], ECFCs [144]

Another model, the OIR model, offers an alternative model to interrogate this and provides significant advantages from both scientific and ethical perspectives. Scientifically, the OIR model is robust and reproducible, providing consistent and measurable endpoints such as pre-retinal neovascularization and revascularization of ischemic areas [145]. These outcomes closely mimic the pathological processes observed in human retinal diseases like diabetic retinopathy, AMD, and retinopathy of prematurity [146,147]. The model has also proven instrumental in understanding the molecular events driven by hypoxia, particularly the role of HIF, and has been pivotal in studying HIF-related targets, including anti-VEGF therapies [136,137,138], heparin derivatives [139], the fascin-1 inhibitor NP-G2-044 [140], multikinase inhibitors like sorafenib [141], and metabolic modulators such as metformin [142] and resveratrol [143]. These studies have expanded the understanding of hypoxia-driven diseases and demonstrated the therapeutic potential of targeting HIF pathways in combination with existing treatments.

From a 3R’s and animal welfare perspective, the OIR model minimizes animal suffering by using controlled oxygen exposure rather than invasive procedures or prolonged distress. This refinement significantly reduces the ethical burden associated with studying hypoxia while maintaining translational relevance. Moreover, the model’s adaptability to various drug delivery methods, including intravitreal and systemic routes, allows for comprehensive evaluations of localized and systemic therapies.

The translational value of the OIR model is further underscored by its role in advancing cell-based therapies, such as endothelial colony-forming cells (ECFCs) [148]. ECFCs were first shown to be efficacious in promoting vascular repair and reducing ischemic damage in the OIR model, where they demonstrated the ability to home to ischemic areas, integrate into damaged vasculature, and promote revascularization [144]. These findings have spurred investigations into the use of ECFCs in other hypoxia-driven conditions, such as diabetic foot ulcers, where localized hypoxia impairs wound healing [149], and MI and reperfusion injury, where ischemic damage is a central feature [150].

A range of neuroprotective and anti-inflammatory agents have been evaluated in the I/R model, reflecting the diversity of pathways that are involved in cell death and inflammation during hypoxic challenge. Edaravone, a free radical scavenger, has shown promise in reducing oxidative stress and promoting cell survival in the IR rat [129]. Metformin, known for its role in diabetes management, has also demonstrated significant protective effects against retinal I/R injury [130]. This is through the promotion of mitochondrial fusion via activation of AMPK, upregulating fusion proteins MFN2 and OPA1, and reducing reactive oxygen species (ROS) generation in RGCs [130]. Peptain-1, an αB-crystallin core-domain-derived peptide, has demonstrated neurovascular protective capabilities in the I/R model by inhibiting pro-inflammatory cytokine-mediated apoptosis to reduce retinal capillary degeneration and neuroinflammation [131]. Another example is the Sirtuin 1 activator resveratrol, which targets cellular energy metabolism pathways and has been shown to protect RGC by modulating oxidative stress and inflammation [132,133,134]. These agents have contributed valuable insights into the role of antioxidants, anti-inflammatory compounds, and neuroprotective agents in combating retinal ischemia–reperfusion injury, advancing research toward therapies that could protect the retina under ischemic stress.

One example therapeutic candidate that has recently emerged in this model is N,N-Dimethyl-3β-hydroxycholenamide (DMHCA) [135]. This compound has gained attention for its ability to modulate inflammatory responses and protect retinal neurons following I/R injury. DMHCA works by inhibiting Ninjurin 1 (Ninj1), a protein involved in promoting inflammation and facilitating cell death during ischemic stress [151]. By targeting Ninj1, DMHCA has shown the potential to reduce both neuronal death and inflammatory response in I/R-induced retinal injury, highlighting its dual-action mechanism as both a neuroprotective and anti-inflammatory agent [135]. Importantly, DMHCA is delivered systemically, which is significant for therapeutic application, as it allows for broader accessibility to retinal tissues via the bloodstream rather than direct ocular administration. Preclinical studies indicate that DMHCA could play an important role in treating retinal ischemic diseases, given its effectiveness in the I/R model. This makes DMHCA a promising candidate for further clinical exploration and illustrates the utility of the I/R model in advancing treatments for ischemic retinal diseases.

Normal hypoxia adaptations are both corrective and protective, designed to either restore normal oxygenation, for example, through VEGF release to direct the formation of new blood vessels, or to reduce the need for oxygen, such as through metabolic changes or in the worst case, cell death [152]. Outcomes after hypoxia often depend on the balance of these two arms, and HIFs are modulated by ROS [153], iron availability [154], and nitric oxide levels [155], among other things. It is also interesting to note, and likely highly pertinent to both preclinical drug evaluation and dosing regimens, that hypoxia is highly interlinked with circadian rhythms [156,157,158], with the balance of the response dictated by the molecular circadian clock. In essence, the hypoxia response elicited in any tissue is dependent on the time of day [159]. This temporal dependence emphasizes the need to take circadian timing into account in both experimental design and therapeutic interventions, as it can greatly affect treatment effectiveness and results.

### 2.4. Neuroprotective Drug Targets

Neurodegenerative diseases are increasingly prevalent in aging populations, with common pathological features including progressive neuronal dysfunction, axonal and dendritic retraction, and neuronal death. These degenerative processes are primarily attributed to mitochondrial dysfunction and neuroinflammation, hallmarks of diseases such as Alzheimer’s disease (AD) and Parkinson’s disease (PD). Pathological hallmarks include toxic protein aggregates like amyloid-beta (Aβ) and tau protein (pTau) in AD [160], and alpha-synuclein (α-syn) and dopaminergic cell loss in the substantia nigra in PD [161]. Microglial cells, the immune cells of the central nervous system (CNS), play a paradoxical role in these diseases. While protective under normal conditions, prolonged activation of microglia triggers excessive inflammatory signaling, exacerbating neuronal damage. Despite extensive research, treatment options remain limited, largely due to challenges in detecting neurodegeneration during early disease stages [162]. Preclinical brain models often fail to capture early pathology, typically focusing on advanced disease stages observable through ex vivo samples or invasive in vivo imaging. Non-invasive imaging techniques for neurodegeneration in the brain remain a significant unmet need.

Preclinical brain models like the MPTP model for PD [163] and APP/PS1 transgenic mice for AD [164] offer critical insights into neurodegenerative mechanisms, but they come with inherent limitations. The MPTP model replicates PD pathology by using a neurotoxin that inhibits mitochondrial complex I, leading to dopaminergic neuron death [165]. Many of these models rely on end-stage pathology, limiting their ability to identify early biomarkers or track disease progression. Imaging techniques such as magnetic resonance imaging (MRI) or positron emission tomography (PET) can provide in vivo insights, but they often lack the spatial resolution or specificity required to detect cellular processes like mitochondrial dysfunction or early apoptosis, which are critical in neurodegeneration. Additionally, these methods are invasive, expensive, and impractical for routine longitudinal studies, creating barriers to studying disease dynamics and drug efficacy over time.

The retina, often referred to as the “window to the brain,” provides a unique and accessible platform for studying neurodegeneration and testing neuroprotective drugs. Retinal tissues share a neuronal network with the brain and exhibit similar pathological changes in neurodegenerative diseases, including reduced dopamine levels, α-syn and amyloid deposits, dopaminergic amacrine cell degeneration, and signs of apoptosis [166]. Importantly, cellular and physiological changes in the retina, such as alterations in RGCs, blood–retina barrier integrity, and microvascular density, mirror the processes observed in the CNS [167]. These parallels make the retina an ideal surrogate for studying CNS diseases. Moreover, imaging tools such as ERG, OCT-A, detection of apoptosing retinal cells (DARC) and fundus imaging provide robust, non-invasive assessments of structural and functional changes, reducing reliance on invasive CNS-focused models and enhancing alignment with the 3R’s principles. These models allow for longitudinal studies, capturing disease progression and therapeutic responses over time with minimal disruption to the subject.

The retina features a complex network of neurons and includes various cell types in both the inner and outer layers. The inner retina consists of RGCs, amacrine cells, bipolar cells, and horizontal cells, while the outer retina is made up of photoreceptors and the RPE [168]. This sophisticated structure positions the retina as an excellent candidate for studies focused on neuroprotective drug targeting and effectiveness, enabling a cell-specific strategy in the development of therapies. These cells face various risk factors that contribute to neurodegeneration, many of which overlap with those affecting the CNS. Common risk factors include oxidative stress, mitochondrial dysfunction, excitotoxicity (glutamate toxicity), neuroinflammation, ischemia, and neurodegenerative diseases [169,170]. Also, each type of inner retinal cell experiences neurodegeneration due to unique stressors. For example, RGCs are particularly vulnerable to increased intraocular pressure in glaucoma, and their degeneration is linked to retinal vein or artery occlusion and microvascular dysfunction [171]. GABAergic or dopaminergic amacrine cells can be influenced by imbalances in neurotransmitters, especially disruptions in the GABA/dopamine system [172]. These differences indicate that targeted neuroprotective strategies are essential to address specific risk factors. For instance, therapies designed to restore the balance of GABA and dopamine may help reduce amacrine cell degeneration. In a similar way, outer retinal cells, such as photoreceptors and RPE, are at risk of neurodegeneration due to factors like light exposure, choroidal vascular insufficiency, lipofuscin accumulation, drusen formation in AMD, and retinal detachment [173,174,175]. Gaining insight into these distinct mechanisms allows for the discovery of more targeted neuroprotective interventions tailored to specific retinal cell types.

Recent technological advancements in retinal imaging have the potential to transform the study of neurodegeneration. The Ocumet Beacon system, currently in development, represents a potential breakthrough in retinal imaging by utilizing mitochondrial flavoprotein fluorescence (FPF), also known as fluorescence lifetime imaging [176]. This innovative, non-invasive imaging tool measures oxidative stress and mitochondrial dysfunction by quantifying the ratio of oxidized to reduced flavoproteins in mitochondria. Unlike spectral-domain optical coherence tomography (SD-OCT), the current gold standard for retinal disease diagnosis and monitoring, which focuses on structural changes, FPF provides insight into subtle functional alterations that may precede visible structural changes [177]. This capability could be particularly valuable for longitudinal studies, enabling researchers to track disease progression and therapeutic responses in real time, and addressing one of the primary challenges in neurodegeneration research.

Complementing this is the Novai system, which focuses on detecting early apoptosis as a biomarker for disease progression [178]. Early apoptosis is a critical event in neurodegenerative diseases, marking the transition from reversible dysfunction to irreversible neuronal loss. By capturing these early cellular changes, the Novai system has the potential to identify disease onset and monitor the efficacy of neuroprotective interventions well before overt symptoms or structural damage occur [178]. Together, the Ocumet and Novai systems provide a comprehensive approach to studying neurodegeneration, from mitochondrial dysfunction to apoptosis, with potential applications in drug development and early diagnosis.

While classically used for studying the brain, existing and new imaging modalities like the Ocumet have led models like MPTP and APP/PS1 mice to be re-examined with a focus on the retina. Retinal changes in the MPTP mouse include RGC loss and neuroinflammation, in addition to the expected dopamine-producing amacrine cell degeneration [165,179]. Imaging studies in this model have shown retinal thinning and delayed oscillatory potential (OP) times in ERG, highlighting retinal dysfunction. Neuroprotective agents like L-DOPA [180] and nicotinamide [181] have demonstrated partial efficacy, with nicotinamide preserving RGCs and mitigating neurodegeneration. Similarly, APP/PS1 and 5XFAD transgenic mouse AD models reveal progressive retinal thinning [182] and the accumulation of Aβ deposits in layers such as the nerve fiber layer (NFL) and inner plexiform layer (IPL) [183]. Imaging techniques, including OCT and fSLO, have detected these changes, mirroring CNS pathology. Functional deficits in these models further support the retina’s utility as a biomarker for AD. These findings emphasize the potential of retinal imaging to detect early neurodegenerative changes non-invasively.

With advancements in retinal imaging and the integration of novel platforms like Ocumet and Novai, the field of neurodegeneration research is poised for significant breakthroughs. These tools not only facilitate the study of disease mechanisms but also improve the development of effective, non-invasive biomarkers and therapeutics, addressing critical unmet needs in diseases like AD and PD. By leveraging retinal indicators of disease, researchers can accelerate the translation of preclinical findings into clinical applications.

## 3. Discussion

Retinal disease models have provided invaluable insights into drug mechanisms, particularly in areas such as inflammation, angiogenesis, hypoxia, and fibrosis, while also serving as essential platforms for evaluating novel gene therapies. Gene therapy for retinal diseases has shown significant promise, with various gene delivery methods demonstrating safety and efficacy in clinical and preclinical studies. The retina’s compartmentalized structure allows targeted delivery to specific cell populations, such as subretinal injections efficiently transducing photoreceptors and the RPE, while intravitreal injections primarily target inner retinal layers. Localized delivery is critical for ensuring effective drug administration while minimizing systemic exposure. Intravitreal injections provide direct access to the vitreous and inner retina, commonly used for gene therapy and biologics such as anti-VEGF treatments, whereas subretinal injections enable more precise targeting of photoreceptors and RPE. Other drug delivery approaches, such as topical administration or systemic supplementation, are less effective due to the blood–retina barrier limiting systemic drug penetration. Advances in sustained-release drug delivery systems, including nanoparticles and gene therapy vectors, are enhancing long-term therapeutic effects and reducing the need for repeated dosing. OCT imaging plays a crucial role in confirming precise drug delivery, ensuring targeted gene therapy administration. Different gene delivery vectors, including AAV2 (Luxturna for RPE65 mutations), AAV8 (RPGR gene therapy for XLRP), and AAV2/8 (PDE6A and PDE6B gene therapy for autosomal recessive RP), have successfully restored gene function and improved visual outcomes [184,185]. For larger genes such as *ABCA4* (Stargardt disease) and *USH2A* (Usher syndrome type 2A), dual-AAV systems have been developed to overcome size limitations, while CRISPR/Cas9-based genome editing has been explored for precise mutation correction. Preclinical models such as rd10 mice (a model of RP) and ABCA4-deficient mice (Stargardt disease) serve as critical platforms for evaluating functional efficacy, with optokinetic response testing assessing visual acuity and ERG measuring photoreceptor function [184]. Functional efficacy is validated using preclinical models such as rd10 mice (a model of RP) and ABCA4-deficient mice (Stargardt disease), where optokinetic response testing assesses visual acuity and ERG measures photoreceptor function [176,186]. These studies highlight the versatility of gene therapy across different retinal compartments and disease models, reinforcing its potential as a model for testing gene delivery systems.

While these studies underscore the versatility of gene therapy in retinal disease models and highlight their potential for evaluating gene delivery systems, their broader translational relevance remains subject to certain limitations. Regulatory agencies often require models that replicate human disease pathology in the directly affected organ, necessitating complementary organ-specific studies. While retinal models share mechanistic similarities with systemic diseases—such as inflammation and fibrosis—their unique immune privilege, neuronal architecture, and BRB characteristics limit their full translational relevance [5]. The BRB, while beneficial in protecting the retina from systemic toxins and pathogens, also presents a significant challenge for drug delivery, as it restricts the systemic penetration of therapeutic agents, necessitating invasive local delivery methods such as intravitreal or subretinal injections. These localized methods provide precise drug administration but have limitations, including the need for repeated dosing, potential intraocular inflammation, and limited diffusion to deeper retinal layers. Topical drug delivery, while non-invasive and patient-friendly, is largely ineffective for retinal diseases due to the poor permeability of large molecules across the corneal and scleral barriers [143]. Similarly, systemic administration often results in subtherapeutic retinal concentrations unless high doses are used, which increases the risk of systemic side effects. Additionally, intravitreal drug delivery results in localized effects, restricting the ability to assess systemic pharmacokinetics, biodistribution, and metabolism, which are crucial for systemic therapeutics. The broader limitations of animal models, including species differences in immune responses, drug metabolism, and receptor expression, further challenge direct translation to humans, highlighting the need for complementary approaches. Variability in non-GLP studies and ethical concerns surrounding animal use also drive the push for alternative methods, such as organoids, microfluidic chips, and computational models. Despite these challenges, retinal models remain particularly useful for studying inflammatory pathways in diseases like diabetic retinopathy, cytokine release in uveitis, and neurodegeneration in glaucoma, Parkinson’s, and Alzheimer’s disease [159,160]. Additionally, ischemic models such as RAO and OIR offer insights into stroke and cardiovascular conditions, while subretinal fibrosis models enable real-time imaging of fibrotic progression [121]. Ultimately, while retinal models serve as robust proof-of-concept platforms, they should be considered complementary rather than standalone models for systemic disease therapeutics, particularly in regulatory settings where organ-specific pathology is required. Integrating in vitro, ex vivo, and computational models alongside retinal models will enhance translational relevance while addressing ethical concerns.

## 4. Conclusions

Retinal models have emerged as invaluable tools in drug development, offering detailed insights into key pathological processes such as inflammation, angiogenesis, hypoxia, and fibrosis. Their unique accessibility and compatibility with advanced imaging technologies allow researchers to study these mechanisms in real time, enabling precise evaluation of therapeutic efficacy and disease progression Beyond their role in ocular research, retinal models provide broader translational relevance, particularly in identifying and validating therapeutic targets for systemic diseases. Furthermore, the retina’s parallels with the CNS provide an opportunity to model neurodegenerative processes, enhancing our understanding of diseases like Alzheimer’s and Parkinson’s. The integration of novel imaging systems, advanced preclinical models, and adherence to ethical principles ensures that retinal research continues to refine predictive accuracy and accelerate the development of innovative treatments for both retinal and systemic conditions. By bridging preclinical findings with clinical needs, these models stand at the forefront of translational science, driving therapeutic innovation across diverse fields.

## 5. Future Directions

Moving forward, the retina is emerging as one of the most critical tissues in the human body, not only for ophthalmic health but as a window into systemic diseases. The rapid growth of Oculomics, driven by advances in machine learning, is transforming the way researchers detect and monitor pathologies across multiple organ systems [187]. While traditional retinal imaging techniques allow researchers to observe structural and functional changes, it is the integration of machine learning with multimodal readouts—such as OCT, OCT-A, fundus imaging, tear analysis, fluorescence angiography, and ERG—that is proving particularly powerful [10,34,188]. This convergence is revolutionizing early disease detection, refining patient stratification for clinical trials, and enabling early interventions with greater precision. The potential of this approach is already evident in clinical research spanning multiple sclerosis, cardiovascular disease, schizophrenia, neurodegenerative disorders, and metabolic disease, among others [187,188,189,190,191,192]. As the value of retinal biomarkers continues to grow, the next step lies in harnessing Oculomics in preclinical research—linking CROs and research institutions with external partners to drive innovation in drug development. This integration could enhance the detection of off-target effects, improve disease monitoring via the eye, and ultimately refine therapeutic strategies across a range of conditions.

## Figures and Tables

**Table 1:** Anti-inflammatory drug targets and their efficacy.

Drug Name	Target Pathway	Mechanism of Action	Retinal Disease Indication	Efficacy and Translational Potential [Ref]
AVD-104 (Aviceda Therapeutics)	Siglec receptors on immune cells (macrophages, microglia)	Glycan-coated nanoparticle targeting Siglecs for modulating inflammation through ITIM signaling	Retinal inflammation, Uveitis	Promising in modulating retinal immune responses [61]
Eculizumab (Soliris)	Complement C5 inhibition	Monoclonal antibody inhibiting C5 to block complement cascade	Initially trialed for AMD, approved for PNH and aHUS	Reducing hemolysis and thrombotic microangiopathy in PNH and aHUS [62]
Ravulizumab (Ultomiris)	Complement C5 inhibition	Long-acting C5 inhibitor	Initially trialed for AMD, approved for PNH and aHUS	Offers extended dosing intervals, for PNH and aHUS [62]
Pegcetacoplan (Empaveli/ Syfovre)	Complement C3 inhibition	C3 inhibitor targeting complement cascade	Initially trialed for AMD, approved for PNH, and geographic atrophy	Approved for PNH and geographic atrophy, strong potential in chronic disease [63]
Teriflunomide	Dendritic cells, Th1/Th17 differentiation, Cytokine production (TNF-α, IFN-γ, IL-17)	Immunosuppressive, reducing Th1/Th17 Differentiation and cytokine production	EAU	Approved for multiple sclerosis and reduces clinical and histopathological EAU scores, promising in retinal models [64]
Apremilast	Th17 cells, Tregs, PI3K/AKT/FoxO1 signaling	Inhibits Th17 and enhances Tregs via the PI3K/AKT/FoxO1 pathway	EAU	Approved for psoriatic arthritis and demonstrates anti-inflammatory effects, helps maintain retinal homeostasis in uveitis [65]
Adalimumab (Humira)	TNF-α inhibition	Blocks TNF-α to reduce chronic inflammation	Uveitis, DME	Approved for RA, Crohn’s disease, potential in retinal conditions like uveitis [66]
Infliximab	TNF-α inhibition	Blocks TNF-α to reduce chronic inflammation	Uveitis, DME	Approved for RA, Crohn’s disease; potential in retinal inflammation [67]
Tocilizumab (Actemra)	IL-6 inhibition	Inhibits IL-6 signaling to reduce inflammatory response	Uveitis, DME	Approved for systemic inflammatory conditions, potential in retinal inflammation [68]
JAK Inhibitors (e.g., Baricitinib)	JAK/STAT pathway	Inhibits Janus kinases (JAKs) to reduce cytokine signaling	Retinal inflammation, Uveitis, DME	Promising in systemic immune disorders, potential in retinal diseases [69]
